# The Ubiquitin Ligase (E3) Psh1p Is Required for Proper Segregation of both Centromeric and Two-Micron Plasmids in *Saccharomyces cerevisiae*

**DOI:** 10.1534/g3.117.300227

**Published:** 2017-09-19

**Authors:** Meredith B. Metzger, Jessica L. Scales, Mitchell F. Dunklebarger, Allan M. Weissman

**Affiliations:** Laboratory of Protein Dynamics and Signaling, Center for Cancer Research, National Cancer Institute, National Institutes of Health, Frederick, Maryland 21702

**Keywords:** ubiquitination, Cse4p, plasmid missegregation, *CEN* plasmid, 2 μm plasmid

## Abstract

Protein degradation by the ubiquitin-proteasome system is essential to many processes. We sought to assess its involvement in the turnover of mitochondrial proteins in *Saccharomyces cerevisiae*. We find that deletion of a specific ubiquitin ligase (E3), Psh1p, increases the abundance of a temperature-sensitive mitochondrial protein, mia40-4pHA, when it is expressed from a centromeric plasmid. Deletion of Psh1p unexpectedly elevates the levels of other proteins expressed from centromeric plasmids. Loss of Psh1p does not increase the rate of turnover of mia40-4pHA, affect total protein synthesis, or increase the protein levels of chromosomal genes. Instead, *psh1Δ* appears to increase the incidence of missegregation of centromeric plasmids relative to their normal 1:1 segregation. After generations of growth with selection for the plasmid, ongoing missegregation would lead to elevated plasmid DNA, mRNA, and protein, all of which we observe in *psh1Δ* cells. The only known substrate of Psh1p is the centromeric histone H3 variant Cse4p, which is targeted for proteasomal degradation after ubiquitination by Psh1p. However, Cse4p overexpression alone does not phenocopy *psh1Δ* in increasing plasmid DNA and protein levels. Instead, elevation of Cse4p leads to an apparent increase in 1:0 plasmid segregation events. Further, 2 μm high-copy yeast plasmids also missegregate in *psh1Δ*, but not when Cse4p alone is overexpressed. These findings demonstrate that Psh1p is required for the faithful inheritance of both centromeric and 2 μm plasmids. Moreover, the effects that loss of Psh1p has on plasmid segregation cannot be accounted for by increased levels of Cse4p.

Post-translational modification of proteins with the 76-amino acid protein ubiquitin targets substrates for a variety of fates. Ubiquitination requires the sequential action of three classes of enzymes: ubiquitin-activating enzyme (E1), a ubiquitin-conjugating enzyme (E2), and one of many different ubiquitin ligases (E3s), which confer exquisite substrate specificity to the process (Zheng and Shabek 2017). Really Interesting New Gene (RING)-type E3s can mediate the transfer of ubiquitin directly from E2 to a substrate, generally onto a substrate lysine residue (Metzger *et al.* 2014; Sundaramoorthy *et al.* 2017). Substrates may be modified with a single ubiquitin or ubiquitin chains. Chains of four or more ubiquitins linked through lysine 48 (K48) of ubiquitin represent the archetypical targeting signal for degradation by the 26S proteasome (Chau *et al.* 1989; Finley *et al.* 1994; Thrower *et al.* 2000). However, it is now evident that other ubiquitin chains can also target substrates for proteasomal degradation (Akutsu *et al.* 2016).

Although the ubiquitin-proteasome system (UPS) directly mediates protein degradation, it can have diverse cellular effects on RNA and DNA. The levels of many mRNAs are affected by UPS-mediated degradation of transcriptional activators or repressors (Yao and Ndoja 2012); one example of this is the degradation of the tumor suppressor p53 by the E3 Mdm2 (Fang *et al.* 2000; Honda and Yasuda 2000). The levels of specific mRNAs can also be affected by cotranslational protein quality control (QC), where monoubiquitination of 40S ribosomal proteins during ribosome stalling leads to degradation of both the mRNA and nascent polypeptide (Doma and Parker 2006; Bengtson and Joazeiro 2010; Juszkiewicz and Hegde 2017; Sundaramoorthy *et al.* 2017). The processes of DNA replication, segregation, and repair are also all known to be regulated by the UPS (Cipolla *et al.* 2016; Garcia-Rodriguez *et al.* 2016; Renaudin *et al.* 2016).

Chromosomal DNA replication and segregation are tightly regulated by cell cycle checkpoints, and errors can have catastrophic effects on cell viability. However, plasmid DNA levels can often be modulated without such effects. In *Saccharomyces cerevisiae*, two types of plasmids commonly used in the laboratory are centromere-containing (*CEN*) plasmids and 2 μm plasmids (Sikorski and Hieter 1989; Christianson *et al.* 1992). Both classes have been engineered to encode selectable marker genes that ensure plasmid maintenance under different selective growth conditions employed in the laboratory. *CEN* plasmids also contain point centromere DNA sequences required for 1:1 equal plasmid segregation into mother and daughter cells and an autonomously replicating sequence (ARS) required for plasmid replication once per cell division in synchrony with chromosome replication (Sikorski and Hieter 1989). These features of the *CEN* plasmid ensure that the plasmid remains, on average, at one copy per yeast cell, although the rate of mitotic loss of *CEN* plasmids is ∼1000 times greater than the rate of chromosome loss (Clarke and Carbon 1980; Hieter *et al.* 1985; Koshland *et al.* 1985; Murray and Szostak 1986; Hegemann *et al.* 1988).

The 2 μm plasmids used for genetic manipulation in yeast contain DNA sequence derived from endogenous 2 μm circles found in the yeast nucleus. This sequence contains an origin of replication and plasmid partitioning elements that enable 2 μm plasmids to be stably maintained (Yen Ting *et al.* 2014). The 2 μm sequence also contains an amplification system, allowing these plasmids to remain at high copy number (∼10–30 copies per cell) uniformly across the population, despite missegregation events (Christianson *et al.* 1992).

In this study, we set out to examine the role of the UPS in QC at yeast mitochondria, but unexpectedly discovered a role for the UPS in plasmid segregation. Loss of a ubiquitin ligase, Psh1p, increases the levels of proteins expressed from *CEN* plasmids without affecting their rates of degradation. Interestingly, we find that Psh1p is required for the proper segregation of both *CEN* and 2 μm plasmids. Loss of Psh1p results in plasmid alterations consistent with an increase in unequal plasmid segregation. This missegregation is distinct from what we observe herein upon overexpression of the only known Psh1p substrate, the centromeric histone H3 variant Cse4p. These results suggest that a previously unappreciated target or function of Psh1p is necessary for proper plasmid segregation.

## Materials and Methods

### Yeast strains, plasmids, and growth conditions

*S. cerevisiae* strains and plasmids are listed in Supplemental Material, Table S1 and Table S2 in File S1, respectively. Cells were cultured at 30° in minimal media supplemented with 2% glucose and the appropriate amino acids, unless otherwise indicated. For experiments requiring galactose induction of *pGAL-FLAG-CSE4*, cells were cultured in 2% raffinose for 12–24 hr prior to the addition of 2% galactose for the indicated time. For spot growth assays, 10-fold serial dilutions beginning with 0.1 OD_600_ units of cells were spotted to minimal media lacking leucine (SC-Leu) and grown at 25° for 3 d, or 37° for 2 d.

### Antibodies

Rabbit polyclonal anti-Cse4p (Pinsky *et al.* 2003) and rabbit polyclonal anti-Sam35p (Chan and Lithgow 2008) were generous gifts from Sue Biggins and Trevor Lithgow, respectively. Commercial antibodies used were: mouse monoclonal Cox1p, mouse monoclonal Hsp60p, mouse monoclonal Porin and rabbit polyclonal Prc1p (all from abcam); mouse monoclonal myc (9E10) and mouse monoclonal green fluorescent protein (GFP; both from Santa Cruz Biotechnology); mouse monoclonal phosphoglycerate kinase (Pgk1p; Life Technologies); and rat monoclonal peroxidase-conjugated anti-HA (3F10; Roche).

### Immunoblotting and cycloheximide chase

Immunoblotting and cycloheximide (CHX) chase analysis were performed as described previously (Metzger *et al.* 2013), with the following changes. Some experiments were carried out at 25 or 37° as indicated. For CHX chases at 37°, cells were cultured at the permissive temperature of 25° until the addition of CHX, at which time the temperature was increased to 37° to accelerate the rate of turnover of the temperature-sensitive (ts-) protein.

### Pulse-chase metabolic labeling and ^35^S incorporation rate

Pulse-chase analyses were performed as described previously (Metzger *et al.* 2013), with the following exceptions: cells were pulsed with ^35^S-labeled methionine/cysteine amino acid mix (EasyTagEXPRESS^35^S Protein Labeling Mix; Perkin Elmer) for 25 min at 25° (for mia40-4pHA or when using strains WCG4a and WCG4-11/21a) or 30 min at 30° (for Fzo1pHA), and cells were chased with 20 mM unlabeled methionine/cysteine at 37° (for mia40-4pHA or when using strains WCG4a and WCG4-11/21a) or 30° (for Fzo1pHA), for the indicated time points. Radioactive signal from three independent experiments was quantified as previously and fit to a one-phase decay curve using GraphPad Prism 7 (GraphPad Software).

To calculate the relative amount of protein that incorporated ^35^S, the radioactive signal at the zero time point was quantified in wild-type (WT) *vs.* *psh1Δ* cells for three independent pulse-chases. The signal was normalized to the signal from WT cells. Prior to immunoprecipitation of mia40-4pHA or Fzo1pHA, an aliquot of total ^35^S-labeled protein lysate was subject to TCA precipitation and counted using a scintillation counter to obtain values for incorporation into total protein. These values were also normalized to the signal from WT cells.

The rate of ^35^S incorporation in WT and *psh1Δ* cells was determined as follows. Cells were grown in minimal media supplemented with leucine, histidine, and uracil at 30° prior to splitting cultures in half. The first half of cells were labeled with EasyTagEXPRESS^35^S Protein Labeling Mix (2 μCi/OD_600_ of yeast cells) and 0.5 μM unlabeled methionine at 30°. These cells were harvested at the time of addition (0 min) and every 10 min after up to 1 hr and then protein isolated like pulse-chase samples. An aliquot of ^35^S-labeled protein was run on an SDS-PAGE gel and the radioactive signal was quantified. At the same time as the ^35^S labeling, 0.5 μM unlabeled methionine was added to the second half of the cells and the OD_600_ was measured at each time point. The radioactive signal was graphed relative to the cell density (OD_600_) at each time point.

### Mitochondrial fractionation and proteinase K treatment

Mitochondria were isolated as described previously (Gregg *et al.* 2009), with the following changes. Cultures were grown in minimal media containing the appropriate amino acids at 25°. Cells were incubated in DTT Buffer (Gregg *et al.* 2009) for 30 min at 25° and in Zymolyase Buffer (Gregg *et al.* 2009) with Zymolyase-100T (MP Biomedicals) for 45 min at 25°, after which time the resulting spheroplasts were washed and resuspended in an equal volume of Zymolyase Buffer without Zymolyase-100T, either at 25° or prewarmed to 37°. CHX (100 μg/ml) was added and spheroplasts were incubated without shaking at 25 or 37° for 1 hr. An aliquot of spheroplasts was removed at this time (“Total”). The remainder were homogenized and fractionated at 12,000 × *g*, as per protocol. A portion of the supernatant resulting from the first 12,000 × *g* spin (“S”) was reserved. The resulting mitochondrial pellet (“P”) was resuspended in SDS-PAGE sample buffer. Total and “S” fractions were precipitated in 10% TCA and washed in acetone prior to resuspending in SDS-PAGE sample buffer. Equivalent proportions of Total, S, and P were analyzed by SDS-PAGE and immunoblotting.

To determine whether mia40-4pHA was mislocalized to the mitochondrial surface, mitochondria isolated as above were resuspended in SM buffer (10 mM MOPS/KOH pH 7.2, 250 mM sucrose). Equivalent amounts were aliquoted to tubes containing either SM buffer alone, SM buffer plus Proteinase K (PrK; 10 μg/ml), SM buffer plus Triton X-100 (Tx-100; 2%), or SM buffer plus both PrK and Tx-100, and incubated for 20 min on ice. Reactions were quenched by the addition of 1× protease inhibitor cocktail (Roche) and PMSF (1 mM), at which time mitochondria were reisolated by spinning at 12,500 × *g*, resuspended in SDS-PAGE sample buffer, and examined by SDS-PAGE and immunoblotting.

### Microscopy

Logarithmically growing yeast cells were immobilized on coverslips coated with conconavalin A and imaged using the 100× objective (1.42 Plan Apo objective with 1.5× magnifying tube lens) of a Nikon Eclipse Ti inverted microscope, equipped with a 6.4 μm-pixel CoolSNAP HQ camera (Photometrics) and Intensilight C-HGFIE illuminator. Z-Sections of 200 nm thick each, spanning a total range of 6 μm, were acquired. ImageJ [National Institutes of Health (NIH)] was used to process the acquired images.

### Real-time PCR (qPCR)

For qPCR analysis of WT and *psh1Δ* strains, six OD_600_ units of cells growing logarithmically at 30° in the appropriate glucose-containing media were harvested and processed. For qPCR analysis of WT, *psh1Δ*, and *pGAL-FLAG-CSE4* strains, two OD_600_ units of cells growing logarithmically at 30° in selective media containing 2% raffinose/2% galactose for 24 hr were harvested and processed. For qPCR on genomic/plasmid DNA, yeast DNA was isolated via glass bead lysis in TSENT buffer (10 mM Tris-HCl pH 8, 100 mM NaCl, 1 mM EDTA, 1% SDS, 2% Tx-100), and phenol/chloroform/isoamyl alcohol (25:24:1), followed by ethanol precipitation of nucleic acids. RNA was removed by RNase A (Thermo Scientific) treatment, followed by reprecipitation of total DNA with ethanol. For reverse transcription (RT)-qPCR from cDNA, RNA was isolated from logarithmically growing cells using the RNeasy Mini Kit (Qiagen) following the protocol for “Purification of Total RNA from Yeast” via an enzymatic lysis with DNase treatment. The iScript Advanced cDNA Synthesis kit (Bio-Rad) was used to synthesize cDNA.

Reactions for qPCR contained 10 ng of genomic/plasmid DNA or cDNA, SsoAdvanced universal SYBR Green supermix (Bio-Rad), and either PrimePCR probes to *ACT1*, *LEU2*, *SEC63*, *ALG1*, *FZO1*, or *MIA40* (Bio-Rad) or primer pairs specific to *FZO1HA*, *mia40-4-HA*, or the pRS315 vector/LEU2 junction, designed according to published recommendations (Nolan *et al.* 2006). Reactions were performed using the PrimePCR cycling program (Bio-Rad) in a CFX96 Touch Real-Time PCR Detection System (Bio-Rad). At least three biological replicates and three technical replicates were done for each yeast strain, as well as the appropriate no RT and template controls. Normalized gene expression data were analyzed using CFX Manager Software with *ACT1* (actin) as the reference target and normalized to the WT strain values. Data were graphed using GraphPad Prism using an unpaired *t*-test to obtain two-tailed *P*-values.

### Plasmid loss assay

Plasmid loss of *CEN* or 2 μm plasmids was measured at 30°. For experiments with WT and *psh1Δ* strains, cells were grown in selective media (SC-Leu) overnight, then an appropriate dilution was plated onto both rich medium (YEPD) and SC-Leu, and plates were incubated for 2–3 d, respectively (0 hr without selection). The remainder of the culture was diluted into YEPD and rediluted after 24 hr. After 48 hr total in nonselective growth media, an appropriate dilution was plated onto YEPD and SC-Leu plates and incubated as above. Colonies were counted and the fraction of plasmid-bearing cells was calculated as the number of colonies on SC-Leu relative to the number of colonies on YEPD for each time point. Three or more biological replicates were performed for each yeast strain. Data were analyzed using GraphPad Prism and an unpaired *t*-test was used to calculate two-tailed *P*-values.

For plasmid loss experiments with *pGAL-FLAG-CSE4* and its isogenic WT and *psh1Δ* strains, cells were grown in 2% raffinose/2% galactose-containing minimal media lacking leucine for 24 hr, then an appropriate dilution was plated onto YEPD and SC-Leu as above (0 hr without selection). The remainder of the culture was diluted into 2% raffinose/2% galactose-containing minimal media with leucine and rediluted after 24 hr. After a total of 48 hr without selection, an appropriate dilution was plated onto YEPD and SC-Leu plates and incubated as above. The fraction of plasmid-bearing cells was calculated and graphed as described above.

### Data availability

Strains, plasmids, and reagents are available upon request.

## Results

### The temperature-sensitive protein mia40-4pHA is an unstable mitochondrial protein

**Figure 1:**
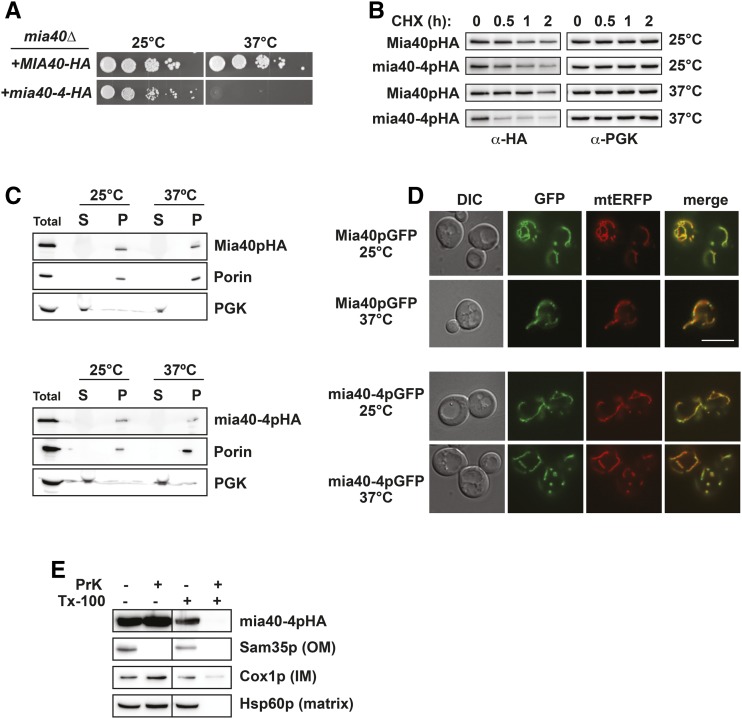
mia40-4pHA is an unstable mitochondrial protein. (A) Ten-fold serial dilutions of *mia40Δ* cells expressing *mia40-4-HA* or *MIA40-HA* from *CEN* plasmids at permissive (25°) or nonpermissive (37°) temperatures were spotted to minimal media lacking leucine. (B) WT yeast were treated with CHX at 25 or 37° for the indicated times to assess the degradation of *CEN* plasmid-expressed mia40-4pHA or Mia40pHA. Proteins were detected by immunoblotting with HA antibody. Phosphoglycerate kinase (PGK) served as a protein loading control. (C) Lysates from cells expressing Mia40pHA (top) or mia40-4pHA (bottom) from *CEN* plasmids at 25 or 37° were fractionated into mitochondrial pellets (P) and postmitochondrial supernatants (S). Fractions were subject to immunoblotting with antibodies to HA, Porin, or PGK. (D) Differential interference contrast (DIC) and fluorescence microscopy at 25 and 37° of cells coexpressing mia40-4pGFP or Mia40pGFP and a mitochondrial matrix-targeted RFP variant, mtERFP. Bar, 10 μm and “merge” is an overlay of GFP and RFP channels. (E) Mitochondria isolated as in (C) were treated with proteinase K (PrK) alone, or in combination with Triton X-100 (Tx-100), and subject to immunoblotting with antibodies specific to the OM protein Sam35p, the IM protein Cox1p, or the matrix protein Hsp60p.

The UPS plays well-characterized and critical roles in protein QC at the endoplasmic reticulum (ER), ribosome, nucleus, and cytosol (Brandman and Hegde 2016; Jones and Gardner 2016; Defenouillere and Fromont-Racine 2017; Preston and Brodsky 2017). Recent studies have revealed a role for the UPS in QC at mitochondria as well (Braun and Westermann 2017), but far less is known regarding the mechanisms or scope of this type of degradation. To begin to explore the determinants of mitochondrial protein turnover, we analyzed a ts- allele of the inner membrane (IM) protein, Mia40p, called *mia40-4* (Chacinska *et al.* 2004). For ease of analysis, we tagged ts- *mia40-4* and WT *MIA40* with the hemagglutinin epitope (HA) and expressed them from *CEN* yeast plasmids. Haploid yeast cells deleted for *MIA40*, expressing only HA-tagged *mia40-4* (*mia40-4-HA*), are inviable at the nonpermissive temperature of 37°, while those expressing WT *MIA40-HA* are not ts- (Figure 1A). To assess protein stability, we performed CHX chase analysis, where the rate of mia40-4pHA turnover was analyzed after cells were treated with CHX to inhibit any new protein synthesis. *CEN* plasmid-expressed mia40-4pHA is unstable at 37° relative to its turnover at the permissive temperature of 25° or the turnover of WT Mia40pHA at either 25 or 37° (Figure 1B).

We next confirmed the proper mitochondrial localization of mia40-4pHA by mitochondrial fractionation. Similar to WT Mia40pHA and the mitochondrial resident protein Porin, mia40-4pHA is found exclusively in the 12,000 × *g* mitochondrial pellet (P) at both permissive and nonpermissive temperatures (Figure 1C). A GFP-tagged version of mia40-4p (mia40-4pGFP) also colocalizes at both temperatures with a mitochondria-targeted enhanced red fluorescent protein (mtERFP) (Scholz *et al.* 2013), nearly identically to WT Mia40pGFP. These data indicate that mia40-4p is found at mitochondria. To confirm that mia40-4pHA is not mislocalized to the outside of mitochondria, mitochondria from cells expressing mia40-4-HA were isolated and treated with proteinase K (PrK). Unlike a control outer membrane (OM) protein (Sam35p), which is subject to proteolysis with PrK treatment alone, mia40-4pHA is only subject to PrK proteolysis when detergent (Tx-100) is also added, similar to control IM (Cox1p) and matrix (Hsp60p) proteins. Thus, mia40-4pHA is a mitochondrially localized, temperature-destabilized protein suitable for QC studies.

### Loss of Psh1p increases levels of mia40-4pHA and Fzo1pHA, without affecting their rates of turnover

**Figure 2:**
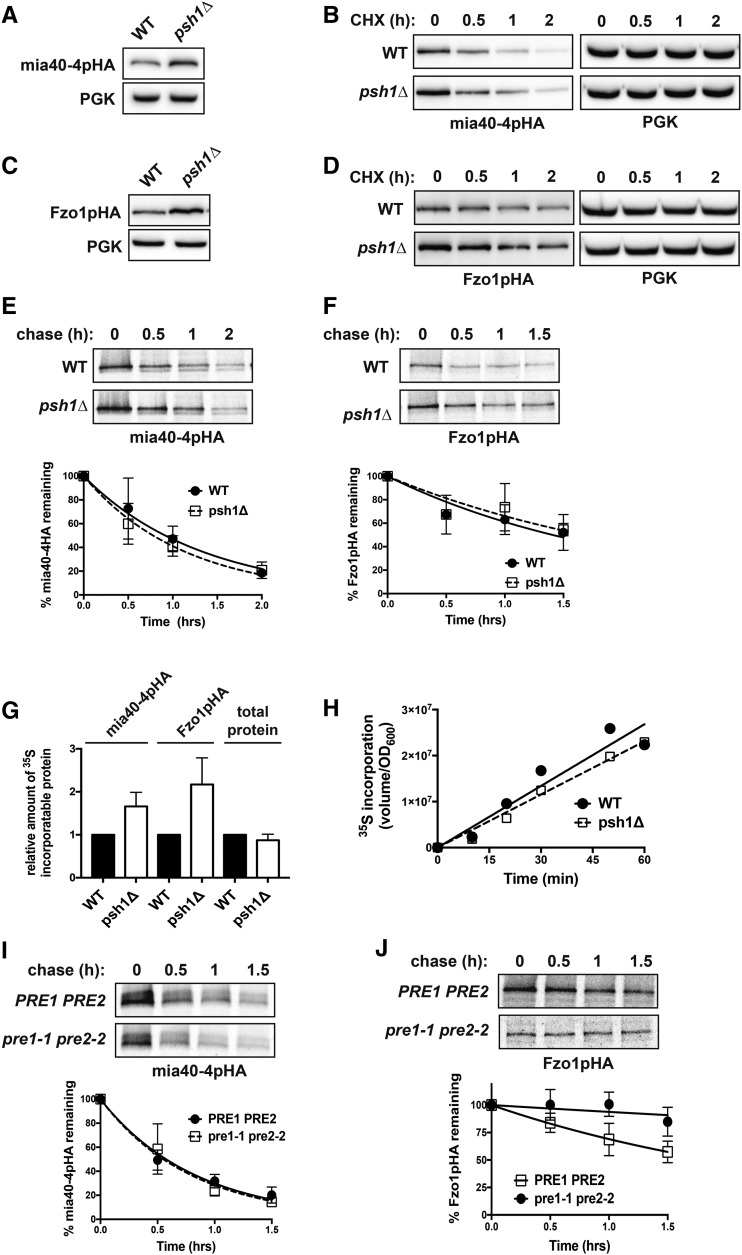
Loss of Psh1p affects the steady-state levels of mia40-4pHA and Fzo1pHA, without affecting their rates of turnover or total cellular protein levels. (A) The steady-state protein level of *CEN* plasmid-expressed mia40-4pHA was assessed at 37° in WT and *psh1Δ* cells by immunoblotting with HA antibody. PGK serves as a control for equal loading. (B) CHX chase for the indicated times assessing turnover of mia40-4pHA expressed from a *CEN* plasmid in WT and *psh1Δ* yeast cells. Proteins were detecting by immunoblotting. (C) The steady-state protein levels of Fzo1pHA, analyzed as in (A) except at 30°. (D) CHX chase of Fzo1pHA, analyzed as in (B) except at 30°. (E) Representative ^35^S pulse-chase analysis to assess turnover of mia40-4pHA in WT and *psh1Δ* cells at 37° at the indicated time points. The mean of three independent experiments is graphed below, with error bars depicting the SD. (F) Representative ^35^S pulse-chase analysis of the turnover of Fzo1pHA in WT and *psh1Δ* at 30° at the indicated time points, analyzed and graphed as in (E). (G) Quantification of ^35^S incorporated into mia40-4pHA, Fzo1pHA, or total protein during a 30-min pulse with ^35^S-labeled methionine/cysteine in *psh1Δ* and WT strains. Values were normalized to the incorporation in the WT strain. The average and SD of three independent experiments is shown. (H) The rate of total protein synthesis in WT and *psh1Δ* strains was measured by analyzing ^35^S methionine/cysteine incorporation into total protein relative to cell density (OD_600_) over time. (I) Representative ^35^S pulse-chase analysis of the turnover of mia40-4pHA in a *pre1-1 pre2*-2 proteasome mutant strain and its *PRE1 PRE2* isogenic WT strain, analyzed and graphed as in (E). (J) Representative ^35^S pulse-chase analysis of the turnover of Fzo1pHA in a *pre1-1 pre2*-2 proteasome mutant strain and its *PRE1 PRE2* isogenic WT strain, analyzed and graphed as in (F).

To identify UPS machinery that might play a role in the temperature-dependent instability of mia40-4pHA, we transformed the *CEN* plasmid expressing mia40-4pHA into a collection of nonessential yeast deletion strains of genes with known or putative roles in the UPS (Ravid and Hochstrasser 2007). We then screened by immunoblotting for deletions that increase the steady-state levels of mia40-4pHA, indicating potential involvement of these genes in its degradation. From this screen, we identified the ubiquitin ligase, Psh1p. Deletion of *PSH1* significantly and reproducibly increases the levels of mia40-4pHA (Figure 2A). Psh1p is a RING-type E3 whose only known substrate is a centromeric histone H3 variant, Cse4p (CENP-A) (Hewawasam *et al.* 2010; Ranjitkar *et al.* 2010). To assess whether the increased levels of mia40-4HA are a consequence of decreased mia40-4pHA degradation in *psh1Δ* cells, we carried out CHX chase experiments (Figure 2B). Accounting for the increased steady-state level of mia40-4pHA in *psh1Δ* cells (Figure 2B, 0 hr), it is not evident that deletion of Psh1p decreases the turnover of mia40-4pHA, which would be expected if Psh1p was an E3 for mia40-4pHA. This led us to assess the effect that loss of Psh1p had on another mitochondrial protein, Fzo1pHA. This mitofusin is an extensively studied UPS substrate targeted for degradation by Mdm30p (Escobar-Henriques *et al.* 2006; Cohen *et al.* 2008) as a consequence of ubiquitination by the E3, SCF^Mdm30p^ (Cohen *et al.* 2008). The steady-state level of Fzo1pHA expressed from a *CEN* plasmid is also increased in *psh1Δ* cells (Figure 2C). However, like mia40-4pHA, CHX chase analysis of Fzo1pHA in *psh1Δ* cells does not show a clear effect on its turnover (Figure 2D, 0 hr).

To quantitatively assess the rate of turnover of mia40-4pHA and Fzo1pHA, we examined their degradation in *psh1Δ* and WT strains by ^35^S pulse-chase metabolic labeling. Quantification of these analyses reveals no significant difference in the rate of degradation of mia40-4pHA (Figure 2E; t_1/2_ = 55 ± 9 and 47 ± 8 min for WT and *psh1Δ*, respectively) or Fzo1pHA (Figure 2F; t_1/2_ = 85 ± 9 and 100 ± 10 min for WT and *psh1Δ*, respectively) in *psh1Δ* compared to WT. However, there is a consistent increase in the amount of ^35^S-labeled mia40-4pHA and Fzo1pHA in the *psh1Δ* strain (Figure 2, E and F, compare 0 hr time points for WT *vs.* *psh1Δ*; quantified in Figure 2G), despite similar rates of growth. This indicates that more mia40-4pHA and Fzo1pHA has been synthesized in *psh1Δ*. Importantly, this is not reflective of an increase in the total radiolabeled protein pool in *psh1Δ* (Figure 2G, total protein), so mia40-4pHA and Fzo1pHA appear to be differentially increased. The rate of total protein synthesis is also similar between the WT and *psh1Δ* strains (Figure 2H).

Since Psh1p does not appear to be targeting mia40-4pHA for degradation, we wondered whether the proteasome was required for its degradation. To analyze this, ^35^S pulse-chase analysis of mia40-4pHA was carried out in a *pre1-1 pre2*-2 proteasome mutant strain (Heinemeyer *et al.* 1991). The rate of turnover of mia40-4pHA was also unchanged in this mutant strain (t_1/2_ = 35 ± 7 and 33 ± 4 min for *PRE1PRE2* and *pre1-1 pre2-2*, respectively). Unlike *psh1Δ*, where there is an increase in the amount of protein synthesized during the pulse with ^35^S-labeled methionine/cysteine, there is actually less protein labeled in the *pre1-1 pre2-2* mutant (Figure 2I, compare 0 hr time points), likely due to the impaired growth characteristic of this strain. The mia40-4pHA protein is also not stabilized by mutations in a vacuolar peptidase (*pep4Δ*; Figure S1A in File S1); in generalized autophagy (*atg5Δ* and *atg8Δ*) or mitophagy machinery (*atg11Δ* and *atg32Δ*; Figure S1B in File S1); or in mitochondrial resident proteases (*yme1Δ*, *afg3Δ*, *yta12Δ*, *oma1Δ*, or *pim1Δ*; Figure S1C in File S1). Having assessed the major cellular proteolytic systems, it remains unclear what is responsible for the instability of mia40-4pHA. Fzo1pHA is stabilized in the proteasome mutant strain (Figure 2J; t_1/2_ = 112 ± 15 and 649 ± 17 min for *PRE1PRE2* and *pre1-1 pre2-2*, respectively), as expected for this well-characterized UPS substrate. There is again no increase in the ^35^S incorporation into Fzo1pHA in the *pre1-1 pre2-2* strain (Figure 2J, 0 hr time points).

### Loss of Psh1p (or Psh1p activity) leads to an increase in the steady-state levels of proteins expressed from CEN plasmids, but not those expressed from the chromosome

**Figure 3:**
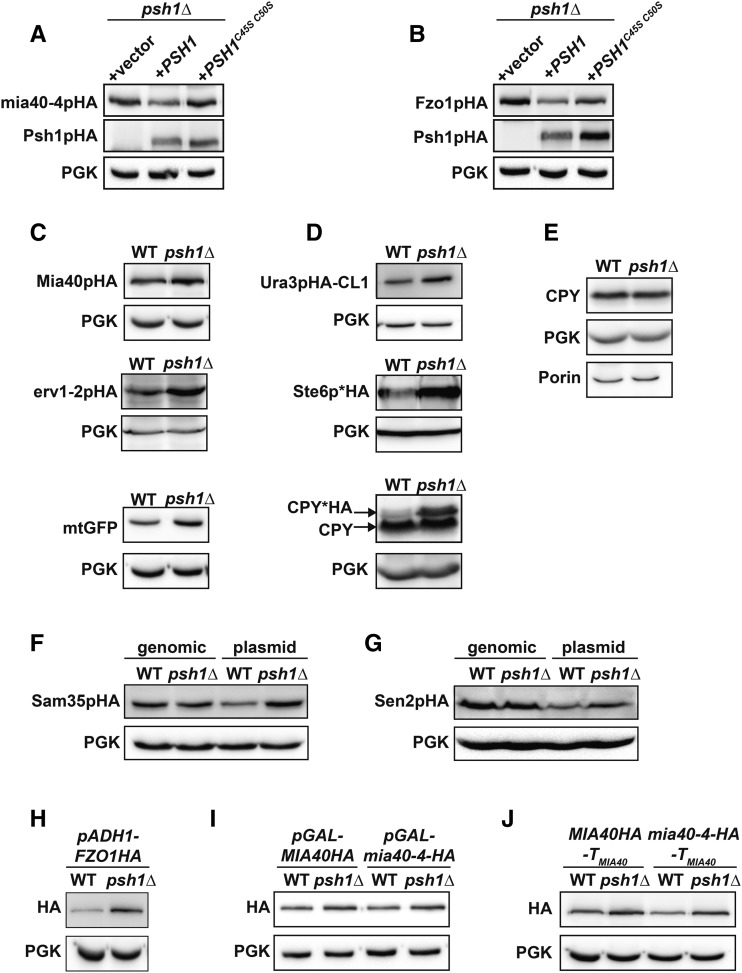
Loss of Psh1p or its ubiquitin ligase activity affects the steady-state levels of many proteins when expressed from plasmids, but not when expressed from the chromosome. (A, B) The steady-state protein levels of *CEN*-plasmid expressed mia40-4pHA (A) or Fzo1pHA (B) were analyzed in *psh1Δ* cells coexpressing either vector, WT *PSH1HA*, or RING domain mutant *PSH1HA*^C45S C50S^ growing at 37° (mia40-4pHA) or 30° (Fzo1pHA) by immunoblotting with HA antibody. Anti-PGK serves as a loading control. (C) Levels of the mitochondrial proteins Mia40pHA, erv1-2pHA, and mitochondrial-targeted GFP (mtGFP) expressed from *CEN* plasmids in WT and *psh1Δ* cells were assessed in WT and *psh1Δ* cells at 30° as in (A). (D) *CEN* plasmid-expressed Ura3pHA-CL1, Ste6p*HA, and CPY*HA were analyzed in WT and *psh1Δ* cells at 30° as in (A) except CPY*HA was visualized using anti-CPY. (E) Chromosomal proteins (CPY, PGK, and Porin) were analyzed in WT and *psh1Δ* cells by immunoblotting using antibodies specific to these targets. (F, G). Sam35pHA (F) or Sen2pHA (G) expressed from either the genome or a *CEN* plasmid were analyzed in WT and *psh1Δ* cells at 30° as in (A). (H–J) *CEN* plasmid-expressed Fzo1pHA under control of the *ADH1* promoter (H) or mia40-4pHA and Mia40pHA under control of the *GAL10* promoter (I) or with the native *MIA40* 3′ untranslated region (J) were assessed in *psh1Δ* and WT cells at 30° (Fzo1pHA) or 37° (mia40-4pHA), as in (A).

We examined whether the ubiquitin ligase activity of Psh1p is required to maintain the normal levels of mia40-4pHA and Fzo1pHA proteins seen in WT cells. While re-expression of WT Psh1p reduces mia40-4pHA and Fzo1pHA in *psh1Δ* cells, a previously described Psh1p RING domain mutant, which lacks E3 activity (*PSH1*^C45S C50S^) (Ranjitkar *et al.* 2010), does not significantly decrease either mia40-4pHA or Fzo1pHA (Figure 3, A and B). This indicates that the E3 activity of Psh1p is required to maintain the normal levels of these proteins seen in WT cells.

We next determined whether loss of Psh1p affected the steady-state levels of other mitochondrial proteins. We expressed WT Mia40pHA, another ts- mitochondrial protein erv1-2pHA (Muller *et al.* 2008), and a mitochondrial matrix-targeted GFP (mtGFP) (Westermann and Neupert 2000) from *CEN* plasmids in WT and *psh1Δ* cells. The levels of all of these proteins are increased when Psh1p is deleted (Figure 3C). We also expressed other UPS substrates, including the cytosolic degron-containing protein Ura3pHA-CL1 (Metzger *et al.* 2008), and the ER-localized misfolded proteins Ste6p*HA and CPY*HA (Hiller *et al.* 1996; Loayza *et al.* 1998), from *CEN* plasmids in *psh1Δ* cells. All show a similar increase in protein abundance in *psh1Δ* (Figure 3D). When assessing CPY* levels using CPY antibody, we noticed that the protein level of endogenous chromosomal CPY is unchanged in *psh1Δ* cells (Figure 3D, CPY). A more detailed analysis of proteins expressed from the genome (CPY, PGK, and Porin) confirms that chromosomal targets are not significantly increased in *psh1Δ* cells relative to WT (Figure 3E).

To more directly assess the effect that loss of Psh1p was having on *CEN*-plasmid *vs.* chromosomal targets, we analyzed the steady-state levels of two mitochondrial proteins (Sam35pHA and Sen2pHA) when expressed from either a *CEN* plasmid or the genome in WT and *psh1Δ* cells. Loss of Psh1p increases the levels of Sam35pHA and Sen2pHA relative to WT cells only when they are expressed from a *CEN* plasmid (Figure 3, F and G). Thus, the increases in protein abundance seen in *psh1Δ* cells are not related to protein location or type, but rather correlate with being expressed from CEN plasmids.

To determine whether the Psh1p-dependent increase in steady-state plasmid protein levels was dependent on the specific promoters or terminators, we replaced the promoters and terminators in our *mia40-4-HA* and *FZO1HA* plasmids used in Figure 1, Figure 2, and Figure 3. *CEN* plasmid-encoded Fzo1pHA was placed under control of the *ADH1* promoter (rather than the *FZO1* promoter) and *CEN* plasmid-encoded mia40-4pHA and Mia40pHA were placed under control of the *GAL10* promoter (rather than the *MIA40* promoter). We also replaced the terminator in our *MIA40HA* and *mia40-4-HA CEN* plasmids with the *MIA40* 3′ untranslated region, rather than *ADH1* terminator used in prior experiments. Regardless of the promoter or terminator, steady-state protein levels of mia40-4pHA, Mia40pHA, and Fzo1pHA all increased in *psh1Δ* cells relative to WT (Figure 3, H–J). Together, these data suggest that *psh1Δ* increases the steady-state levels of proteins when they are expressed from *CEN* plasmids and not when they are expressed from the genome. This effect is a consequence of loss of the ubiquitin ligase activity of Psh1p, and is independent of the promoter or terminator used.

### Loss of Psh1p increases the mRNA of plasmid-expressed genes due to higher plasmid DNA levels resulting from plasmid missegregation

**Figure 4:**
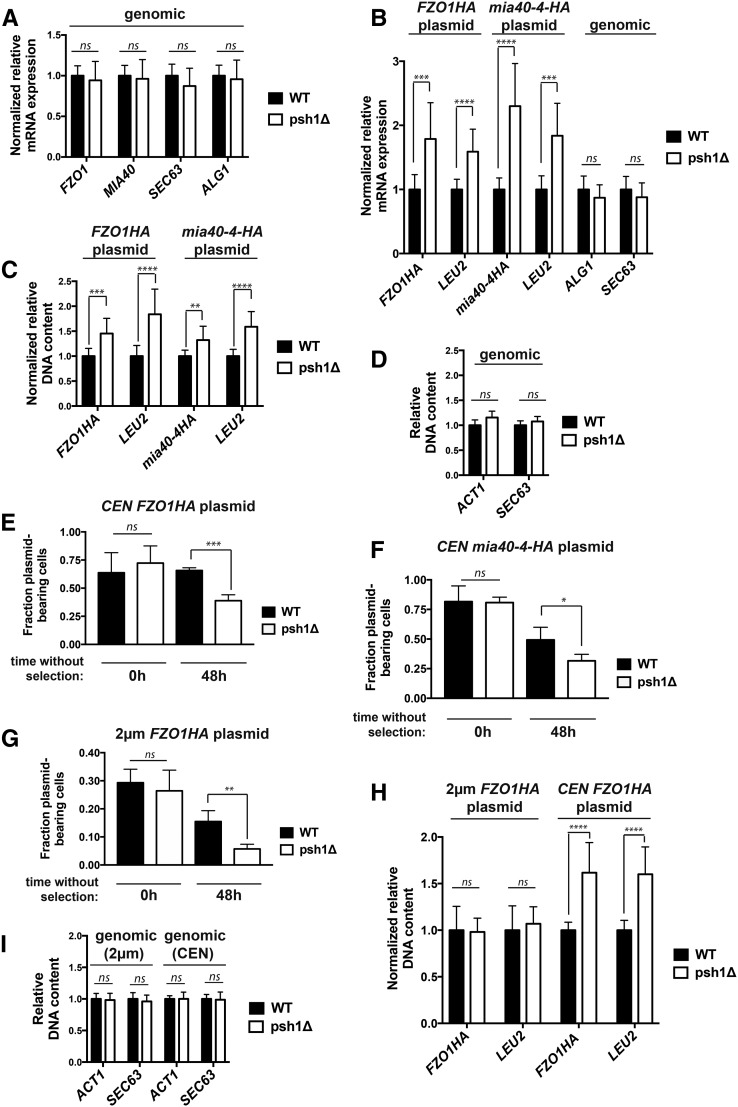
Loss of Psh1p increases mRNA levels of genes expressed from *CEN* plasmids due to higher plasmid DNA content achieved through plasmid missegregation. (A) Normalized mRNA expression of the endogenous, genome-expressed genes *FZO1*, *MIA40*, *SEC63*, or *ALG1* in WT and *psh1Δ* cells grown in YPD medium, not expressing a plasmid, was determined using RT-qPCR analysis. Expression was normalized to *ACT1* levels and is graphed relative to WT cells; error bars represent the SD of at least three biological replicates with three technical replicates each. (B) Normalized mRNA expression of *FZO1HA*, *mia40-4HA*, and *LEU2* in WT and *psh1Δ* cells carrying the indicated *CEN* plasmid, and normalized mRNA expression of genomic *SEC63* and *ALG1* in WT and *psh1Δ* cells expressing the *CEN FZO1HA* plasmid were determined as in (A). (C) Normalized DNA levels of *CEN* plasmid *FZO1HA*, *mia40-4HA*, and *LEU2* in DNA harvested from WT and *psh1Δ* cells carrying the indicated *CEN* plasmid as determined by qPCR on DNA samples. Plasmid DNA content was normalized to the genomic level of *ACT1* and graphed as in (A). (D) DNA levels of genome-expressed *ACT1* and *SEC63* in the DNA used in (C) from cells expressing the *FZO1HA CEN* plasmid as determined by qPCR. Values are graphed relative to WT cells; error bars represent the SD of at least three biological replicates with three technical replicates each. (E–G) The fraction of *FZO1HA CEN* (E) *mia40-4HA CEN* (F) or *FZO1HA* 2 μm plasmid-bearing WT and *psh1Δ* cells after 0 and 48 hr without selection for the plasmid was assessed by comparing the number of cells able to grow on selective media to the cell number on YPD. Error bars represent the SD of at least three biological replicates. (H) Normalized DNA levels of 2 μm and *CEN* plasmid *FZO1HA* and *LEU2* in WT and *psh1Δ* cells, determined as in (C). (I) DNA levels of genome-expressed *ACT1* and *SEC63* in the DNA used in (H) as determined by qPCR, graphed and analyzed as in (D). For (A–I) *P*-values are calculated from a two-tailed *t*-test and * *P* < 0.05, ** *P* < 0.01, *** *P* < 0.001, **** *P* < 0.0001, and ns = *P* > 0.05.

We next assessed whether the increase in *CEN* plasmid-derived protein in *psh1Δ* cells results from an increase in mRNA levels of the genes in question. Quantitative reverse transcription qPCR (RT-qPCR) with primers specific to genomic-expressed *FZO1*, *MIA40*, *SEC63*, and *ALG1* shows no difference in the mRNA levels of WT and *psh1Δ* strains when cells are grown in YPD medium and not expressing plasmid (Figure 4A; normalized to *ACT1*). Similarly, there is no change in the relative mRNA levels of chromosomal *ALG1* and *SEC63* in WT and *psh1Δ* cells expressing the *CEN FZO1HA* (Figure 4B; genomic) or *CEN* mia40-4pHA (data not shown) plasmid and growing in medium lacking leucine. In contrast, the mRNA levels of HA-tagged *FZO1HA* and *mia40-4-HA* expressed from these *CEN* plasmids are significantly increased in *psh1Δ* cells relative to WT (Figure 4B; normalized to *ACT1*). We also see similar increases in the mRNA levels of *LEU2*, the selective marker gene on these plasmids required for their maintenance (and deleted from the chromosome of the yeast strain used here).

Since protein levels are increased by loss of Psh1p even when different promoter and terminators are used (Figure 3, H–J), we hypothesized that the observed increases in mRNA levels did not result from changes in transcription or mRNA stability, but instead from increased plasmid DNA content in *psh1Δ* strains. Analysis by qPCR reveals that the DNA levels of *CEN* plasmid *FZO1HA*, *mia40-4-HA*, and *LEU2* are indeed increased in a *psh1Δ* strain relative to WT (Figure 4C; normalized to *ACT1* levels). The DNA levels of genomic targets (*ACT1* and *SEC63*) in these same *psh1Δ* cells expressing *CEN FZO1HA* (Figure 4D) or mia40-4pHA (data not shown) are unaffected. Taken together, these data indicate that loss of Psh1p increases *CEN* plasmid DNA content, thus leading to an increase in the mRNA and proteins encoded by these plasmids.

The increase in *CEN* plasmid DNA levels in the population of cells seen when Psh1p is absent could be resulting from an increased incidence of unequal plasmid segregation (*i.e.*, nondisjunction) during mitosis. This could initiate with 2:0 missegregation events following normal *CEN* plasmid replication, such that there is a net increase in plasmid DNA in one cell with a concurrent complete loss in the other. Previous experiments where we measure increased *CEN* plasmid DNA, mRNA, and protein levels in *psh1Δ* cells were performed under conditions where cells were grown in selective media lacking leucine to maintain the *CEN* plasmids. Any cells that lose *CEN* plasmid through missegregation would not be maintained in the population because they now lack the selective marker gene on the plasmids, and are inviable in selective media. Cells with one or more copies of the *CEN* plasmid would continue to replicate the plasmid and divide normally, thus leading to an increase in the overall plasmid DNA content within the population of cells over time.

To examine whether the increased plasmid DNA levels measured in a population of *psh1Δ* cells under selective conditions could be resulting from unequal plasmid segregation, we measured plasmid loss in *psh1Δ* cells. The plasmid loss assay measures the fraction of cells maintaining plasmid after growth without selection, as assessed by the presence of plasmid-encoded *LEU2* marker (see also Figure 7 and legend). WT and *psh1Δ* cells expressing *CEN FZO1HA* plasmid were first grown in selective media, where there is no significant difference in the percentage of WT and *psh1Δ* cells that contain at least one copy of the *CEN* plasmid (Figure 4E, 0 hr). Cells were then grown in nonselective media for 48 hr, after which they were again plated on selective media; ∼65% of WT cells retain the *CEN FZO1HA* plasmid after 48 hr without selection, compared to <40% of *psh1Δ* cells (Figure 4E, 48 hr). Using the same assay, we see a similar enhanced loss of *CEN mia40-4-HA* plasmid (Figure 4F) or *CEN* empty vector plasmid (Figure S2A in File S1, compare WT and *psh1Δ*, 48 hr) in *psh1Δ* cells. This reduction in plasmid-bearing cells, in conjunction with the increase in overall plasmid DNA content when Psh1p is absent, suggests that there is indeed an increased incidence of unequal *CEN* plasmid segregation.

We wished to extend these findings to noncentromeric plasmids, so we next examined 2 μm plasmids. High-copy number, 2 μm plasmids appear to use a chromosome-coupled segregation mechanism, although the exact mechanism mediating this “hitchhiking” is unclear, and the fidelity of plasmid segregation is less than for *CEN* plasmids (Sau *et al.* 2015). Interestingly, after 48 hr without selection, only 6% of *psh1Δ* cells retain a 2 μm plasmid encoding *Fzo1HA* with a *LEU2* selectable marker (Figure 4G), while 16% of WT cells do so. Expression of a 2 μm *mia40-4-HA* plasmid negatively affects cell viability (data not shown), precluding its inclusion in this analysis, but a 2 μm empty vector plasmid is lost similarly to the 2 μm *FZO1HA* plasmid (Figure S2B in File S1, compare WT and *psh1Δ*, 48 hr). Even with increased 2 μm plasmid loss, and in contrast to the *CEN* plasmids, we do not detect a change in the amount of 2 μm plasmid DNA in a *psh1Δ* strain compared to WT under selective media conditions (Figure 4H). The DNA levels of chromosomal genes are again also unaffected by *psh1Δ* in cells expressing the *CEN* or 2 μm *FZO1* plasmids (Figure 4I). In agreement with unchanged 2 μm plasmid DNA content, the levels of 2 μm plasmid-derived proteins are also not increased by loss of Psh1p (Figure S2, C and D in File S1). The lack of a change in 2 μm DNA or protein levels is perhaps not surprising, as 2 μm plasmids are known to buffer changes in their levels by amplification using a recombination-induced rolling circle replication mechanism (Chan *et al.* 2013). Regardless, the fidelity of segregation of both *CEN* and 2 μm plasmids appears to be affected by loss of Psh1p.

### Overexpression of the Psh1p degradation target Cse4p does not phenocopy loss of Psh1p in increasing CEN plasmid-derived protein levels

**Figure 5:**
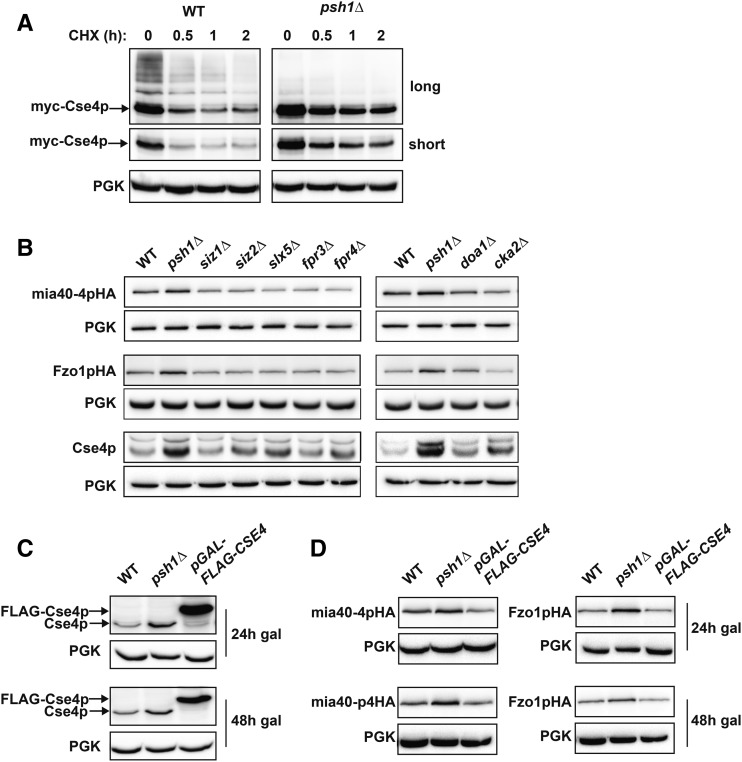
Overexpression of Cse4p does not phenocopy loss of Psh1p in increasing *CEN* plasmid protein levels. (A) CHX chase for the indicated times assessing turnover of myc-Cse4p in WT and *psh1Δ* yeast cells. Proteins were detecting by immunoblotting with myc antibody. Both long and short exposures are shown. Anti-PGK serves as a loading control. (B) The steady-state protein levels of *CEN* plasmid-expressed mia40-4pHA and Fzo1pHA, as well as endogenous genome-expressed Cse4p, were detected by immunoblotting with HA or Cse4p antibodies, in the indicated deletion strains. (C) Immunoblotting for Cse4p using Cse4p antibodies in WT cells, *psh1Δ* cells, or cells overexpressing Cse4p from the genome (*pGAL-FLAG-CSE4*) for the indicated time points after continuous galactose induction. (D) Immunoblotting using anti-HA for *CEN* plasmid-expressed mia40-4pHA or Fzo1pHA as in (C).

The only known target of Psh1p is the centromeric histone H3 variant Cse4p (CENP-A). Several elegant studies have demonstrated that Psh1p ubiquitinates Cse4p that is not properly localized to the centromere, targeting this mislocalized pool of Cse4p for degradation (Hewawasam *et al.* 2010; Ranjitkar *et al.* 2010). Ultimately, this ensures that Cse4p is exclusively centromere-restricted, which is required for proper chromosome segregation (Hajra *et al.* 2006; Au *et al.* 2008). Loss of Psh1p decreases the rate of turnover of endogenous Cse4p at all stages of the cell cycle (Ranjitkar *et al.* 2010). We confirm that our *psh1Δ* strain stabilizes Cse4p and, in darker exposures, we see higher molecular weight laddering that is suggestive of ubiquitination of Cse4p in WT cells, but is absent in *psh1Δ* cells (Figure 5A).

Since Cse4p misregulation leads to chromosome transmission defects (Morey *et al.* 2004; Au *et al.* 2008), we wondered whether the increased Cse4p levels in *psh1Δ* cells could also be affecting *CEN* plasmid segregation and ultimately leading to increased *CEN* plasmid-derived protein levels. As Cse4p is essential for viability (Stoler *et al.* 1995), it cannot be deleted from *psh1Δ* cells, preventing us from directly asking whether it is necessary for the plasmid missegregation phenotypes. However, other proteins have been implicated in increasing the levels of Cse4p either through modulation of Psh1p activity toward Cse4p or by affecting Cse4p levels independently of Psh1p. We assessed mia40-4pHA and Fzo1pHA in deletions of these genes. These include mutants in sumoylation machinery (Siz1p, Siz2p, and Slx5p), proline isomerases (Fpr3p and Fpr4p), a factor required for ubiquitin homeostasis (Doa1p), and casein kinase 2 (Cka2p) (Au *et al.* 2013; Hewawasam *et al.* 2014; Ohkuni *et al.* 2014, 2016; Canzonetta *et al.* 2015). Deletion of an F-box protein, Rcy1p, also affects Cse4p levels (Cheng *et al.* 2016); this deletion was not assessed due to a severe growth defect. For most of these deletions, there is a significant increase in endogenous Cse4p relative to WT cells (Figure 5B; Cse4p). For deletions where Cse4p is unaltered, differences in strain background or growth conditions, or that we assessed endogenous Cse4p, may all be potential explanations. Importantly, the steady-state protein levels of *CEN* plasmid-based mia40-4pHA and Fzo1pHA do not significantly change in any of the deletion strains that manifest increased Cse4p (Figure 5B). This is in contrast to what is observed with *psh1Δ* (Figure 5B).

To assess the effects of elevated Cse4p more directly, we used a well-characterized Cse4p overexpression system in WT cells to ask whether this could phenocopy *psh1Δ* cells. This strain contains a chromosomal, galactose-inducible FLAG-tagged Cse4p (*pGAL-FLAG-CSE4*) (Ranjitkar *et al.* 2010). The pattern of occupancy of Cse4p at the centromere is perturbed similarly by either *psh1Δ* or by FLAG-Cse4p overexpression using this strain (Hildebrand and Biggins 2016). Cse4p is increased above WT levels by either deletion of Psh1p or overexpression of FLAG-Cse4p for 24 or 48 hr (Figure 5C). Despite the substantial increase in Cse4p levels with FLAG-Cse4p overexpression, the protein levels of mia40-4pHA and Fzo1pHA are largely unchanged (Figure 5D). This is in contrast to the significant increase that we observe with *psh1Δ*. Thus, increasing Cse4p to varying levels, using either previously characterized deletion strains or induced overexpression, does not phenocopy *psh1Δ* in increasing *CEN* plasmid-derived protein levels.

### Overexpression of Cse4p does not affect plasmid DNA levels or loss in the same manner as loss of Psh1p

**Figure 6:**
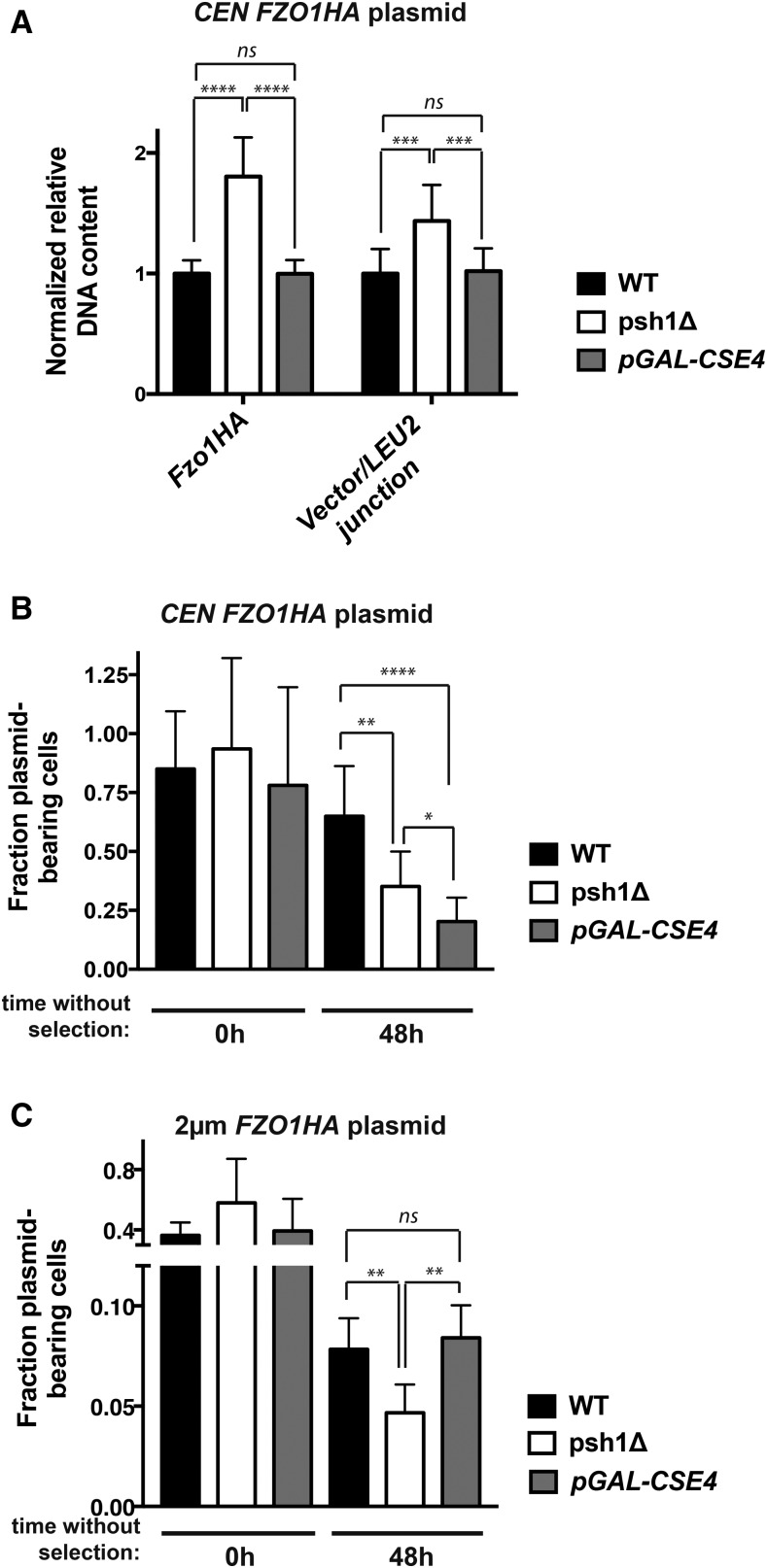
Overexpression of Cse4p increases *CEN*, but not 2 μm, plasmid loss and does not affect plasmid DNA content under selective growth conditions. (A) Normalized DNA levels of *CEN FZO1HA* plasmid were detected by qPCR using two plasmid-specific primer pairs (*FZO1HA* and vector/*LEU2* junction) on DNA harvested from WT cells, *psh1Δ* cells, or cells overexpressing Cse4p from the chromosome (*pGAL-FLAG-CSE4*) for 24 hr in selective media. *LEU2* primers could not be used, as the mutated *leu2-3*,*112* allele is present in the genome of these strains. DNA content was normalized to *ACT1* levels and graphed as in Figure 4C. See Figure S3A in File S1 legend for further detail. (B) The fraction of *CEN FZO1HA* plasmid-bearing was analyzed as in Figure 4D after 0 and 48 hr growth in media without selection for the *CEN* plasmid. Cells were grown in medium containing galactose for 24 hr prior to the shift to nonselective medium, and galactose induction was continued during growth without selection. Error bars represent the SD of at least three biological replicates. (C) The fraction of 2 μm *FZO1HA* plasmid-bearing WT cells, *psh1Δ* cells, or cells overexpressing Cse4p from the chromosome (*pGAL-FLAG-CSE4*) was analyzed as in (B) For (A–C) *P*-values are calculated from a two-tailed *t*-test; * *P* < 0.05, ** *P* < 0.01, *** *P* < 0.001, **** *P* < 0.0001, and ns = *P* > 0.05.

To confirm that elevated Cse4p is alone insufficient to mediate the effects we see in *psh1Δ*, we reassessed *CEN FZO1HA* plasmid DNA levels by qPCR after FLAG-Cse4p overexpression. Some decrease in the amplification efficiency of genomic targets is observed (Figure S3A in File S1), perhaps related to reported Cse4p-mediated genomic instability (Morey *et al.* 2004; Au *et al.* 2008). *CEN FZO1HA* plasmid DNA levels, in marked contrast to *psh1Δ*, are unchanged by FLAG-Cse4p overexpression (Figure 6A; *Fzo1HA* and Vector/*LEU2* junction-normalized to *ACT1*). The lack of an increase in plasmid DNA is consistent with the relative protein levels in Figure 5D. These results indicate that, unlike loss of Psh1p, the direct overexpression of its substrate Cse4p does not result in czhanges consistent with unequal plasmid missegregation.

We next repeated the plasmid loss assay in cells where FLAG-Cse4p was overexpressed. As expected, loss of Psh1p results in increased *CEN FZO1HA* plasmid loss under nonselective growth conditions (Figure 6B, *psh1Δ*). Interestingly, cells overexpressing FLAG-Cse4p lose this *CEN* plasmid to an even greater extent than *psh1Δ* (Figure 6B, *pGAL-FLAG-CSE4*). Similar results are seen for a *CEN* plasmid without insert (Figure S2A in File S1). The levels of *CEN* plasmid DNA and protein from Cse4p-overexpressing cells are not elevated when they are grown under selection (Figure 5D and Figure 6A), which is distinct from the increases seen in *psh1Δ* cells under the same conditions. This suggests that the plasmid loss resulting from elevation of Cse4p alone is mechanistically distinct from that observed with loss of Psh1p. In addition to plasmid loss by missegregation of replicated plasmid (*e.g.*, 2:0 plasmid missegregation), loss can also occur via a 1:0 mechanism. In this case, plasmid does not properly replicate or is destroyed before cell division; such a mechanism may be at play here for Cse4p-overexpressing cells.

In addition to binding to centromeric DNA, Cse4p has been reported to bind at low levels to the *STB* DNA locus of 2 μm plasmids (Hajra *et al.* 2006; Huang *et al.* 2011), perhaps playing a role in the hitchhiking segregation of this type of plasmid. Mutation of Cse4p also affects *STB*-containing reporter plasmid segregation (Hajra *et al.* 2006). Since *psh1Δ* leads to increased 2 μm plasmid loss, we wondered whether this was mediated by Psh1p’s effects on Cse4p levels or, like *CEN* plasmids, is different. Thus, we examined 2 μm *FZO1HA* plasmid loss under nonselective galactose-induction conditions in WT, *psh1Δ*, and FLAG-Cse4p overexpressing cells. We again see 2 μm plasmid loss when Psh1p is absent under these growth conditions. In contrast to the *CEN* plasmid, however, overexpression of Cse4p alone using *pGAL-FLAG-CSE4* does not lead to increased 2 μm plasmid loss (Figure 6C), representing a striking dichotomy with *psh1Δ*. Similar results are seen with a 2 μm vector lacking insert (Figure S2B in File S1). These data argue against a direct role for Cse4p in 2 μm plasmid loss. Taken together, the distinct patterns of *CEN* and 2 μm plasmid missegregation and *CEN* plasmid DNA levels seen in *psh1Δ* cells strongly suggest a non-Cse4p-mediated effect of Psh1p on plasmid DNA maintenance.

## Discussion

**Figure 7:**
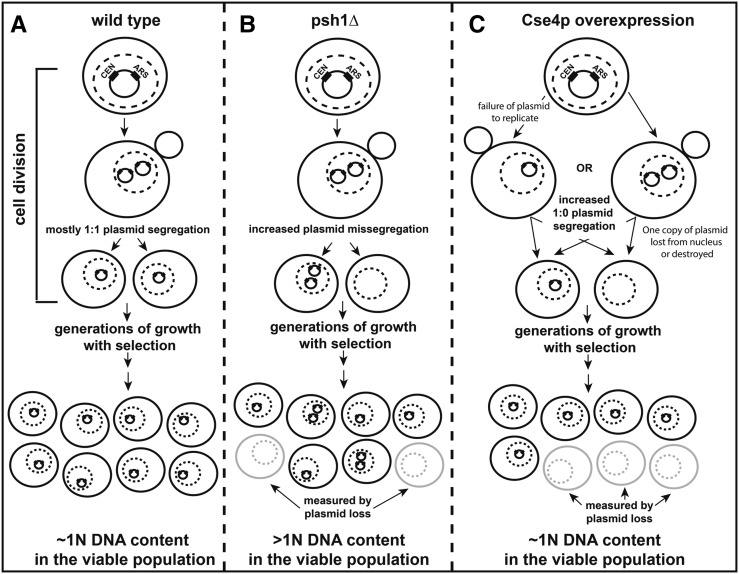
Models for the distinct mechanisms of plasmid missegregation in haploid *psh1Δ* and Cse4p-overexpressing cells. (A) In WT cells, *CEN*/ARS-containing yeast plasmids generally accurately replicate and segregate in a 1:1 manner during cell division, and thus have a low frequency of plasmid loss. After generations of growth in media selective for cells containing the plasmid, cells typically maintain one copy of the plasmid and are viable. Thus, the overall plasmid DNA content in the population remains close to 1N in haploid yeast cells. (B) In haploid *psh1Δ* cells, *CEN*/ARS-containing plasmids are replicated, but have an apparent increased propensity for missegregation. One cell may receive two copies of the plasmid, while the other receives none. Under selective growth conditions, cells without plasmid are inviable. Yeast with one or more copies of plasmid would be viable and continue to divide, leading to an overall increase in the DNA content (>1N) in the population after generations of growth with selection for the plasmid. Shown is a model for the simplest 2:0 missegregation of plasmid, but other mechanisms of missegregation (*i.e.*, 4:0, 3:1, etc.) also become possible after initiating missegregation events have occurred. An increase in cells lacking plasmid is detected by the plasmid loss assay, which measures colony forming units on selective *vs.* nonselective media after generations of growth without selection for the plasmid. (C) In cells overexpressing Cse4p, there appears to be an increase in the incidence of 1:0 segregation of *CEN*/ARS-containing plasmids. This could occur because the plasmid fails to replicate properly, or if one copy of the plasmid is lost from the nucleus or degraded following replication. Regardless, the net result is that only one progeny cell receives a copy of plasmid. Again, the cell without plasmid would be inviable under selective growth conditions and this loss would be measured by the plasmid loss assay. The cell with one copy of the plasmid would remain viable and continue to divide, with the DNA content in the population remaining close to 1N after generations of growth in medium selective for the plasmid. For (A–C) the nucleus is indicated by a dotted line.

In this study, we demonstrate that loss of the ubiquitin ligase Psh1p increases the *CEN* plasmid DNA content in cells grown under selective pressure. This in turn elevates plasmid-encoded mRNA and protein. The net increase in plasmid DNA is coupled with a higher incidence of plasmid loss from cells that have undergone multiple rounds of division without selective pressure. This suggests that the *CEN* plasmid is missegregating more frequently in *psh1Δ* than in WT cells, where a 1:1 equal plasmid segregation generally occurs (Figure 7, A and B, shown is the simplest mechanism—ongoing 2:0 unequal plasmid segregation—but, as plasmid copy number increases in some cells, other permutations of missegregation are also possible, if not likely). Thus, the *CEN* plasmid is likely replicating properly but there is increased missegregation during mitosis. Ongoing missegregation would lead to some population of cells containing more than one copy of the plasmid, which increases the overall steady-state plasmid DNA content of the population. A similar plasmid missegregation could be responsible for the recently reported plasmid loss in a diploid *psh1Δ* strain, which accompanies loss of heterozygosity (Herrero and Thorpe 2016). Interestingly, while overexpression of Cse4p—the only known target of Psh1p—leads to even greater plasmid loss than *psh1Δ*, their mechanisms are distinct. As there is no significant increase in *CEN* plasmid DNA levels when Cse4p is overexpressed under selective conditions, this suggests an increase in an apparent 1:0 mechanism of segregation wherein plasmid fails to replicate prior to mitosis or one copy of the replicated plasmid is lost from the nucleus or destroyed (Figure 7C). The ARS and CEN DNA sequences are in close proximity on the plasmid, and Cse4p overexpression is known to lead to centromere spreading to proximal regions (Hildebrand and Biggins 2016), which could feasibly interfere with plasmid replication. Alternatively, the genomic instability observed with Cse4p overexpression (Morey *et al.* 2004; Au *et al.* 2008) could be increasing the rate of formation of dicentric plasmids, which break during DNA segregation and lead to plasmid instability and loss (Mann and Davis 1983). This 1:0 mechanism of plasmid loss has been observed to occur at greater than twice the frequency of 2:0 events (Koshland *et al.* 1985), making the increased missegregation seen with loss of Psh1p that much more significant.

The differences in plasmid DNA, mRNA, and protein levels between *psh1Δ* and Cse4p overexpression strongly suggest that the dysregulation of a distinct target or function of Psh1p is required to mediate these *CEN* plasmid missegregation events. The established *pGAL-FLAG-CSE4* overexpression system elevates Cse4p levels more than *psh1Δ* does, making it difficult to completely exclude Cse4p dosage effects. However, direct overexpression of FLAG-Cse4p or elevated levels of Cse4p from loss of Psh1p result in similar increases in the centromeric distribution of this histone H3 variant (Hildebrand and Biggins 2016), suggesting that, at least with respect to centromere loading of Cse4p, the two are equivalent. We cannot rule out other differences between the two conditions, but, for several reasons, we favor a model where the loss of ubiquitination of a novel target of Psh1p is a requisite component of the missegregation mechanism. Varying the length of time of galactose induction of FLAG-Cse4p, and thus increasing the number of rounds of division the cells have undergone, has no impact on *CEN* plasmid protein levels. Similarly, modulating Cse4p through gene deletions is without effect. Further, if Psh1p was a dedicated ubiquitin ligase for only Cse4p, one might reasonably expect that Psh1p would only be located where Cse4p is found, with the exception of the *CEN* localization of Cse4p, where it is protected from Psh1p-mediated ubiquitination and degradation. Rather, there is evidence that Psh1p localizes to kinetochores independently of Cse4p. Fractionation of micrococcal nuclease-treated chromatin reveals that Cse4p distributes almost equally between the soluble fraction and the insoluble pellet, while Psh1p remains entirely in the insoluble pellet, where the larger kinetochore protein complex fractionates (Hewawasam *et al.* 2010). Also, deletions that result in Cse4p being mislocalized to locations other than centromeres do not change the kinetochore association of Psh1p.

Whether there is a *bona fide* target of Psh1p at kinetochores, and what role Psh1p may play in its regulation, is an area for future investigation. Ubiquitin ligases rarely have a single substrate. However, one of the challenges in the field is the comprehensive identification of their substrates due to their transient and low-affinity substrate interactions. It will be important to identify Psh1p substrates other than Cse4p with the goal of understanding more completely the means by which this ligase affects not only plasmid, but potentially chromosome, inheritance. *CEN* plasmids are lost at least 100 times more frequently than a normal chromosome (Hieter *et al.* 1985; Koshland *et al.* 1985), making it unsurprising that we see no gross effect on genomic targets in *psh1Δ* cells. Also, chromosomal loss events would likely be deleterious to haploid cells, causing genomic instability and, in most cases, loss of viability. We and others see no measurable difference in the rate of growth or cell cycle progression between WT and *psh1Δ* cells (data not shown) (Ranjitkar *et al.* 2010; Herrero and Thorpe 2016), which would indicate relatively subtle effects on chromosome missegregation. Loss of Psh1p has been shown to modulate kinetochore protein stoichiometry (Herrero and Thorpe 2016) with a resultant increase in genomic instability phenotypes (Yuen *et al.* 2007). However, while increased loss of a particular genomic locus was seen in *psh1Δ* cells in three independent genetic screens (Yuen *et al.* 2007; Herrero and Thorpe 2016), loss of an entire artificial chromosome fragment was not observed (Yuen *et al.* 2007). In contrast, elevation of Cse4p or its orthologs lead to genomic instability and chromosome missegregation and loss (Morey *et al.* 2004; Heun *et al.* 2006; Au *et al.* 2008; Amato *et al.* 2009; Gonzalez *et al.* 2014), suggesting that, as is the case herein with plasmids, at the level of the genome, there are functional distinctions between deleting this ubiquitin ligase and its known target, Cse4p.

With respect to 2 μm plasmid loss, we see a clear difference between *psh1Δ* and Cse4p overexpression. Cse4p overexpression does not increase 2 μm plasmid loss, while deletion of Psh1p does, again arguing that the two are having distinct cellular effects. The precise mechanism of segregation of 2 μm plasmids is not clear, but one model is that they “hitchhike” along with chromosomes during mitosis (Yen Ting *et al.* 2014; Sau *et al.* 2015). Thus, it is possible that 2 μm missegregation occurs because Psh1p is indeed having effects on chromosome segregation. Another possibility is that Psh1p plays a direct role in 2 μm plasmid segregation independent of its role in *CEN* plasmid or chromosome segregation. Interestingly, a study describing Ubiquitin-Activated Interaction Traps (UBAITs) technology, which can identify proteins in proximity to specific ubiquitin ligases, used Psh1p as a bait and identified a component of the 2 μm plasmid partitioning system (O’Connor *et al.* 2015). This raises the intriguing possibility that Psh1p may be in close proximity to 2 μm plasmids. Sumoylation of 2 μm plasmid-associated proteins has been previously demonstrated to be required for plasmid maintenance (Pinder *et al.* 2013), so it is conceivable that other post-translational modifications like ubiquitin may also play a role.

The original goal of this study was to identify and characterize UPS components acting on mitochondrial QC substrates. This is still an open and active area of investigation. While the mia40-4pHA ts- protein is unstable at the nonpermissive temperature, we do not find it to be stabilized in the *pre1-1 pre2-2* mutant strain by pulse-chase under these conditions. Interestingly, a previous study showed that steady-state levels of mia40-4p are increased by treatment with the proteasome inhibitor, MG132, but not by a proteasome mutant strain (Bragoszewski *et al.* 2013). The turnover of mia40-4pHA is also not affected by mutations in autophagy or mitochondrial protease genes, including Pim1p (Lon) and the mAAA- and iAAA-proteases. The mechanism of mia40-4pHA degradation remains unclear and warrants continued investigation.

Finally, this study raises a cautionary note in interpreting increased levels of plasmid-expressed mRNA and protein. Plasmids are a powerful tool to assess transcription and post-transcriptional regulation. Our work underscores that perturbation of cellular factors can lead to unexpected and unobvious alterations in plasmid levels and the genes they encode.

## Supplementary Material

Supplemental material is available online at www.g3journal.org/lookup/suppl/doi:10.1534/g3.117.300227/-/DC1.
