# Supplementary material for: The Ubiquitin Ligase (E3) Psh1p Is Required for Proper Segregation of both Centromeric and Two-Micron Plasmids in Saccharomyces cerevisiae
Source: G3 (Bethesda). 2017 Sep 19;7(11):3731–43. doi: 10.1534/g3.117.300227 (PMC5677152; doi:10.1534/g3.117.300227)
Supplement: Supplementary file 1 [file 3731FileS1.docx]

**Supplemental Information for:**

**The ubiquitin ligase (E3) Psh1p is required for proper segregation of both centromeric and two micron plasmids in *Saccharomyces cerevisiae***

Meredith B. Metzger, Jessica L. Scales, Mitchell F. Dunklebarger, and Allan M. Weissman

| **Table S1.** Yeast strains used in this study | | |
| --- | --- | --- |
| **Strain** | **Relevant genotype** | **Source** |
| SBY3 | *ura3-1 leu2-3,112 his3-11 trp1-1 can1-100 ade2-1 bar1-1* | (Ranjitkar *et al.* 2010) |
| SBY8336 | *ura3-1 leu2-3,112 his3-11 trp1-1 can1-100 ade2-1 bar1-1 psh1::KanMX* | (Ranjitkar *et al.* 2010) |
| SBY8904 | *ura3-1 leu2-3,112 his3-11 trp1-1 can1-100 ade2-1 bar1-1*  *pGAL-FLAG-CSE4::URA3* | (Ranjitkar *et al.* 2010) |
| WCG4-11/21a | *ura3-5Δ leu2-3,112 his3-11,15 pre1-1 pre2-2* | (Heinemeyer *et al.* 1991) |
| WCG4a | *ura3-5Δ leu2-3,112 his3-11,15 PRE1 PRE2* | (Heinemeyer *et al.* 1991) |
| BY4741 | *his3Δ leu2Δ ura3Δ met15Δ* | GE Dharmacon |
| yMM36 | *his3Δ leu2Δ ura3Δ met15Δ SAM35HA::KanMX* | This study |
| yMM40 | *his3Δ leu2Δ ura3Δ met15Δ SEN2HA::KanMX* | This study |
| yMM106 | *his3Δ leu2Δ ura3Δ met15Δ psh1::KanMX* | This study |
| yMM118 | *his3Δ leu2Δ ura3Δ met15Δ mia40::KanMX [CEN LEU2 MIA40-HA]* | This study |
| yMM119 | *his3Δ leu2Δ ura3Δ met15Δ mia40::KanMX [CEN LEU2 mia40-4-HA]* | This study |
| yMM152 | *his3Δ leu2Δ ura3Δ met15Δ psh1::HIS6MX SEN2HA::KanMX* | This study |
| yMM156 | *his3Δ leu2Δ ura3Δ met15Δ psh1::HIS6MX SAM35HA::KanMX* | This study |
| *siz1Δ* | *his3Δ leu2Δ ura3Δ met15Δ siz1::KanMX* | GE Dharmacon |
| *siz2Δ* | *his3Δ leu2Δ ura3Δ met15Δ siz2::KanMX* | GE Dharmacon |
| *slx5Δ* | *his3Δ leu2Δ ura3Δ met15Δ slx5::KanMX* | GE Dharmacon |
| *fpr3Δ* | *his3Δ leu2Δ ura3Δ met15Δ fpr3::KanMX* | GE Dharmacon |
| *fpr4Δ* | *his3Δ leu2Δ ura3Δ met15Δ fpr4::KanMX* | GE Dharmacon |
| *doa1Δ* | *his3Δ leu2Δ ura3Δ met15Δ doa1::KanMX* | GE Dharmacon |
| *cka2Δ* | *his3Δ leu2Δ ura3Δ met15Δ cka2::KanMX* | GE Dharmacon |
| *pep4Δ* | *his3Δ leu2Δ ura3Δ met15Δ pep4::KanMX* | GE Dharmacon |
| *atg5Δ* | *his3Δ leu2Δ ura3Δ met15Δ atg5::KanMX* | GE Dharmacon |
| *atg8Δ* | *his3Δ leu2Δ ura3Δ met15Δ atg8::KanMX* | GE Dharmacon |
| *atg32Δ* | *his3Δ leu2Δ ura3Δ met15Δ atg32::KanMX* | GE Dharmacon |
| *atg11Δ* | *his3Δ leu2Δ ura3Δ met15Δ atg11::KanMX* | GE Dharmacon |
| *yme1Δ* | *his3Δ leu2Δ ura3Δ met15Δ yme1::KanMX* | GE Dharmacon |
| *afg3Δ* | *his3Δ leu2Δ ura3Δ met15Δ afg3::KanMX* | GE Dharmacon |
| *yta12Δ* | *his3Δ leu2Δ ura3Δ met15Δ yta12::KanMX* | GE Dharmacon |
| *oma1Δ* | *his3Δ leu2Δ ura3Δ met15Δ oma1::KanMX* | GE Dharmacon |
| *pim1Δ* | *his3Δ leu2Δ ura3Δ met15Δ pim1::KanMX* | GE Dharmacon |

| **Table S2.** Yeast plasmids used in this study | | |
| --- | --- | --- |
| **Plasmid** | **Relevant genotype** | **Source** |
| pMM157 | *CEN LEU2 pSAM35-SAM35HA* | This study |
| pMM160 | *CEN LEU2 pSEN2-SEN2HA* | This study |
| pMM162 | *CEN LEU2 pMIA40-MIA40-HA-T_ADH1_* | This study |
| pMM164 | *CEN LEU2 pMIA40-mia40-4-HA-T_ADH1_* | This study |
| pMM174 | *CEN LEU2 erv1-2-HA* | This study |
| pMM190 | *CEN LEU2 pFZO1-FZO1HA* | This study |
| pMM200 | *CEN LEU2 pGAL-MIA40-HA-T_ADH1_* | This study |
| pMM201 | *CEN LEU2 pGAL-mia40-4-HA-T_ADH1_* | This study |
| pMM205 | *CEN LEU2 pMIA40-MIA40-HA-T_MIA40_* | This study |
| pMM206 | *CEN LEU2 pMIA40-mia40-4-HA-T_MIA40_* | This study |
| pMM230 | *2μ LEU2 pFZO1-FZO1HA* | This study |
| pMM236 | *CEN HIS3 pPSH1-PSH1HA-C45S C50S* | This study |
| pMD6 | *CEN LEU2 pMIA40-MIA40-GFP* | This study |
| pMD8 | *CEN LEU2 pMIA40-mia40-4-GFP* | This study |
| pMD12 | *CEN URA3 pTPI-mtERFP* | This study |
| pMD17 | *CEN HIS3 pPSH1-PSH1HA* | This study |
| SB816 | *2μ URA3 pGAL-myc-CSE4* | (Ranjitkar *et al.* 2010) |
| pADH1-Fzo1pHA | *CEN URA3 pADH1-FZO1HA* | (Cohen *et al.* 2008) |
| ZKb085 | *CEN URA3 CPY*HA* | This study |
| pSM1911 | *2μ URA3 pPGK-STE6*HA* | (Huyer *et al.* 2004) |
| pSM2288 | *CEN LEU2 URA3HA-CL1* | (Metzger *et al.* 2008) |
| pYX142-mtGFP | *CEN LEU2 pTP1-mtGFP* | (Westermann and Neupert 2000) |
| pRS315 | *CEN LEU2* | (Sikorski and Hieter 1989) |
| ­­­­pRS425 | *2μ LEU2* | (Christianson *et al.* 1992) |

**Supplemental Figure Legends**

**Supplemental Figure S1. mia40-4pHA is not degraded by the vacuole, autophagy, or mitochondrial resident proteases.** The turnover of mia40-4pHA was assessed by cycloheximide (CHX) chase at 37° for the indicated times when expressed from a *CEN* plasmid in strains mutant for the vacuolar peptidase, Pep4p (A), autophagy machinery (B; Atg5p, Atg8p, Atg32p, and Atg11p), or mitochondrial resident proteases (C; Yme1p, Afg3p, Yta12p, Oma1p, and Pim1p). Proteins were detecting by immunoblotting with anti-HA.

**Supplemental Figure S2. *CEN* and 2μm plasmids are affected differently by Cse4p over-expression.** (A) The fraction of *CEN* empty vector plasmid (pRS315)-bearing WT cells, *psh1Δ* cells, or cells overexpressing Cse4p from the chromosome (*pGAL-FLAG-CSE4*) was analyzed as in Figure 6B after 0 and 48 hrs growth in media without selection for the *CEN* plasmid. Cells were grown in media containing galactose for 24 hrs prior to the shift to non-selective media and galactose induction was continued during growth without selection. Error bars represent the SD of at least 3 biological replicates. P-values are calculated from a two-tailed t-test and **= P-value < 0.01, * = P-value < 0.05, and ns = P-value > 0.05. (B) The fraction of 2μm empty vector plasmid (pRS425)-bearing WT cells, *psh1Δ* cells, or cells overexpressing Cse4p from the chromosome (*pGAL-FLAG-CSE4*) was analyzed as in A. (C,D) The steady-state protein levels of *CEN* and 2μm-plasmid expressed Fzo1pHA (C) or Mia40pHA (D) were analyzed in WT and *psh1Δ* cells growing at 30° by immunoblotting with HA antibody. Anti-PGK serves as a loading control.

**Supplemental Figure S3. Genomic DNA qPCR amplification when Cse4p is overexpressed.** (A) The DNA levels of genome-expressed *ACT1*, *SEC63*, *ALG1*, and *MIA40* as assessed by qPCR amplification of DNA harvested from WT cells, *psh1Δ* cells, or cells overexpressing Cse4p from the chromosome (*pGAL-FLAG-CSE4*) for 24 hrs. All three strains were grown in the same galactose-containing media, no gross differences in the integrity of the DNA were seen (as assessed by agarose gel), and the total amount of DNA added to the qPCR reactions was similar. Values are graphed relative to the amplification in WT cells; error bars represent the SD of at least three biological replicates with three technical replicates each. P-values are calculated from a two-tailed t-test and ****= P-value <0.0001, **= P-value < 0.01, and ns = P-value > 0.05.

**Supplemental References**

Christianson, T. W., R. S. Sikorski, M. Dante, J. H. Shero and P. Hieter, 1992 Multifunctional yeast high-copy-number shuttle vectors. Gene 110**:** 119-122.

Cohen, M. M., G. P. Leboucher, N. Livnat-Levanon, M. H. Glickman and A. M. Weissman, 2008 Ubiquitin-proteasome-dependent degradation of a mitofusin, a critical regulator of mitochondrial fusion. Mol Biol Cell 19**:** 2457-2464.

Heinemeyer, W., A. Simeon, H. H. Hirsch, H. H. Schiffer, U. Teichert *et al.*, 1991 Lysosomal and non-lysosomal proteolysis in the eukaryotic cell: studies on yeast. Biochem Soc Trans 19**:** 724-725.

Huyer, G., W. F. Piluek, Z. Fansler, S. G. Kreft, M. Hochstrasser *et al.*, 2004 Distinct machinery is required in Saccharomyces cerevisiae for the endoplasmic reticulum-associated degradation of a multispanning membrane protein and a soluble luminal protein. J Biol Chem 279**:** 38369-38378.

Metzger, M. B., M. J. Maurer, B. M. Dancy and S. Michaelis, 2008 Degradation of a cytosolic protein requires endoplasmic reticulum-associated degradation machinery. J Biol Chem 283**:** 32302-32316.

Ranjitkar, P., M. O. Press, X. Yi, R. Baker, M. J. MacCoss *et al.*, 2010 An E3 ubiquitin ligase prevents ectopic localization of the centromeric histone H3 variant via the centromere targeting domain. Mol Cell 40**:** 455-464.

Sikorski, R. S., and P. Hieter, 1989 A system of shuttle vectors and yeast host strains designed for efficient manipulation of DNA in Saccharomyces cerevisiae. Genetics 122**:** 19-27.

Westermann, B., and W. Neupert, 2000 Mitochondria-targeted green fluorescent proteins: convenient tools for the study of organelle biogenesis in Saccharomyces cerevisiae. Yeast 16**:** 1421-1427.
